# Bioactive Exopolysaccharides Reveal *Camellia oleifera* Infected by the Fungus *Exobasidium gracile* Could Have a Functional Use

**DOI:** 10.3390/molecules24112048

**Published:** 2019-05-29

**Authors:** Zhe Dong, Wen Liu, Dejian Zhou, Peipei Li, Teng Wang, Kunlai Sun, Yuqin Zhao, Jie Wang, Bin Wang, Yin Chen

**Affiliations:** 1 College of Food and Pharmacy, Zhejiang Ocean University, 1 South Haida Road, Zhoushan 316000, China; DZ18868006001@163.com (Z.D.); yxlliuwen@outlook.com (W.L.); 18368082615@163.com (D.Z.); m18368090226@163.com (T.W.); 2 Marine School, Ningbo University, 818 Fenghua Road, Ningbo 315000, China; Lipeipei1985@163.com; 3 Zhejiang Mariculture Research Institute, 28 Tiyu Road, Zhoushan 316000, China; 4 Zhejiang Provincial Engineering Technology Research Center of Marine Biomedical Products, 1 South Haida Road, Zhoushan 316000, China; sunqinlai@126.com (K.S.); zhaoy@zjou.edu.cn (Y.Z.); w19900101wangjie@163.com (J.W.); wangbin4159@hotmail.com (B.W.)

**Keywords:** *Camellia oleifera*, *Exobasidium gracile*, exoplysaccharide, immunoregulatory activity

## Abstract

*Camellia oleifera* is an important Chinese commercial crop. *Camellia oleifera* can display abnormal leaves due to infection by the parasitic fungus *Exobasidium gracile*. *Exobasidium gracile* was isolated from infected leaves and used in fermentation, and exopolysaccharides EP0-1 and EP0.5-1 were purified from the fermentation broth. EP0-1 was an alkaline polysaccharide consisting mainly of the linkages α-d-Manp(1→, →2)-α-d-Manp(1→ and →6)-α-d-Manp(1→, →3)-α-d-Glcp(1→ and→4)-α-d-Glcp(1→, terminal β-d-Galf, (1→5)-β-d-Galf, and terminal β-D-GlcN(1→. EP0.5-1 was an acidic galactofuranose-containing polysaccharide. It contained the linkages of α-d-Manp(1→, →2)-α-d-Manp(1→, →6)-α-d-Manp(1→,→2, 6)-α-d-Manp(1→, →4)-α-d-Glcp(1→, and →4)-α-d-GlcUA(1→. Galactofuranose linkages were composed of terminal β-d-Galf, (1→6)-β-d-Galf and (1→2)-β-d-Galf. *Exobasidium gracile* exopolysaccharides displayed significant immunoregulatory activity by activating macrophages. This research indicates that infected leaves from *Camellia oleifera* including the exopolysaccharides produced by the parasitic fungus *Exobasidium gracile* by are worth further investigation as a functional product.

## 1. Introduction

*Camellia oleifera* Abel. is an important oil crop distributed abundantly in the South of China [1]. In April, the increase in temperature and abundant rainfall increases the growth rate of new leaves. During growth, some leaves display abnormal development. Abnormal leaves are swollen and thicker than healthy leaves, showing rolling of margins and they are spongy inside. A film also forms on the leaf surface and the color changes from green to ivory. Abnormal leaves are popular due to their sweet and refreshing taste, and in the Hunan Province of China, people use abnormal leaves as a health food [2,3].

A recent study revealed that the abnormal development of *C. oleifera* leaves is caused by the parasitic fungus *Exobasidium gracile,* which belongs to the basidiomycetous genus *Exobasidium* [4]. The genus *Exobasidium* contains more than 170 species worldwide that are plant pathogens that cause leaf lesions, leaf and flower galls, leaf blight, or a deformity known as “witches’ broom”. The hosts of *Exobasidium* fungi are predominantly from four plant families: Ericaceae, Escalloniaceae, Theaceae and Symplocaceae. *Exobasidium gracile* is a representative of some members of *Exobasidium*, and is a parasite of *Camellia* species. It can damage commercial tea plants and thus has economic importance. *Camellia oleifera* is rich in China and has a huge cultivation area. Among tea varieties, black tea is a widely consumed beverage. Processing steps for black tea involve fermentation. After fermentation, the content of acidic heteropolysaccharides increase, accompanied by higher antioxidant and α-glucosidase inhibitory activities [5,6,7]. Herein, using analysis of black tea polysaccharides, variations in sugar composition were studied to provide insight into the infected leaves from *C. oleifera* and their value for health promoting properties [8]. Previous study showed infected leaves extract had better antioxidant activities and contained more total sugars, reducing sugars, crude polysaccharides, and total phenol content than uninfected leaves (Supplementary Figure S1 and Table S1). Infected leaf extract and crude polysaccharides showed greater antioxidant activity than those from healthy leaves. Healthy leaves mainly contained glucose, rhamnose, galactose, and arabinose, which are similar to tea polysaccharides. Infected leaves contained more mannose, glucose and galactose, but less arabinose (Supplementary Table S1), which indicates that crude polysaccharides from infected leaves contain fungal polysaccharides.

The infected leaves of *Camellia oleifera* which were used as food contained abundant polysaccharides, including exopolysaccharides produced by the infecting fungus. Tea polysaccharides were proved to be a kind of bioactive polysaccharides. The fungal polysaccharides in the infected could also have some function. If so, both the infected leaves of *Camellia oleifera* and the fungus *E. gracile* can be used as functional foods. To investigate the fungal polysaccharide part of the polysaccharides in the infected leaves and explores the fungi resources for functional use, *Exobasidium gracile* was isolated from the infected leaves of *C. oleifera* and the exopolysaccharides were isolated and studied in this paper.

## 2. Results and Discussion

### 2.1. Fermentation of Fungus, and Extraction and Purification of Fungal Exopolysaccharides

**Figure 1:**
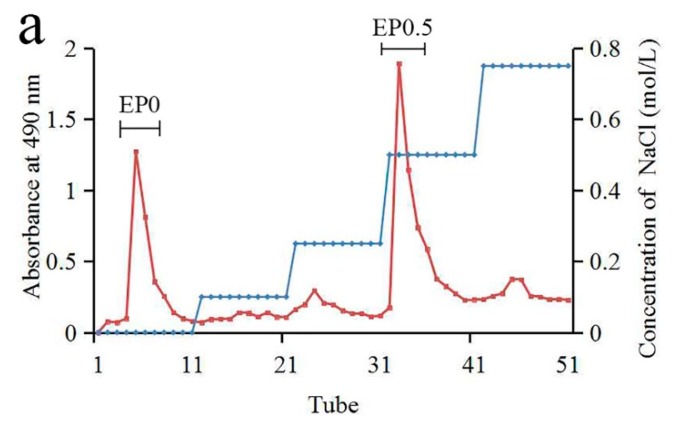

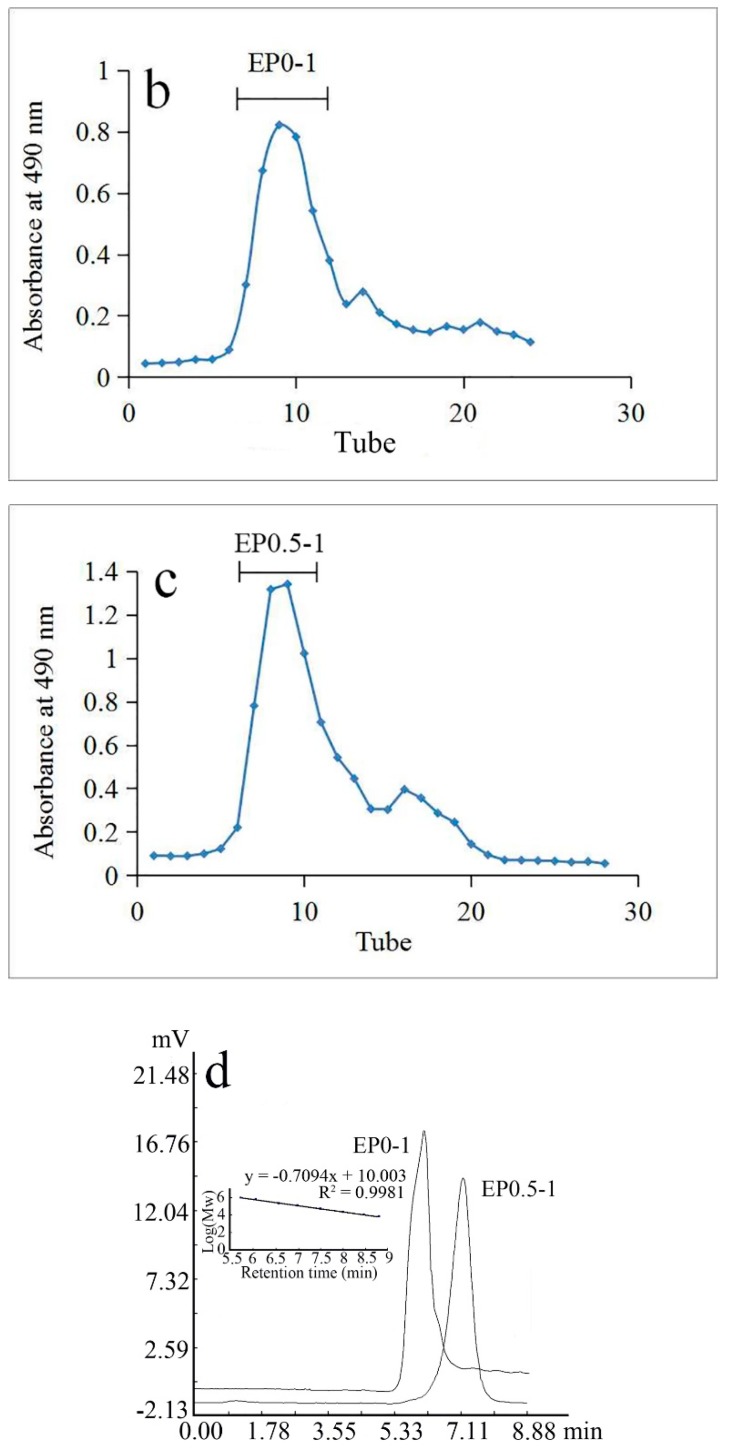
****** Isolation and HPGPC chromatogram of the polysaccharides produced by *Exobasidium gracile*. (**a**) The crude polysaccharides were fractionated by anion exchange chromatography through a Q Sepharose Fast Flow column and isolated to two fraction EP0 and EP0.5. (**b**) The major fractions eluted by water and 0.5 mol/L NaCl were further purified on a Sephacryl S-200 column and isolated a fraction EP0-1. (**c**) The major fractions eluted by water and 0.5 mol/L NaCl were further purified on a Sephacryl S-200 column and isolated a fraction EP0.5-1. (**d**) The purity and molecular weights of EP0-1 and EP0.5-1 were studied using HPGPC.

Our previous data indicated that the concentration of polysaccharides in infected leaves was increased and they could contain large amounts of exopolysaccharides. To explore the use of infected leaves as a functional product, the exopolysaccharides must inevitably be considered. To study the exopolysaccharides individually, the fungus *E. gracile* was isolated from *C. oleifera*, purified and used in fermentation. The yield of the crude polysaccharide from the fermentation broth of *E. gracile* was 4.53%. The crude polysaccharides were fractionated by anion exchange chromatography through a Q Sepharose Fast Flow column (Figure 1a). The major fractions eluted by water and 0.5 mol/L NaCl were further purified on a Sephacryl S-200 column and named as EP0-1 and EP0.5-1, respectively (Figure 1b,c). The yield of EP0-1 from crude polysaccharide was approximately 45% and that of EP0.5-1 was approximately 30%.

### 2.2. Analysis of Physicochemical Characteristics

The purity and molecular weights of EP0-1 and EP0.5-1 were studied using HPGPC. Both produced a single symmetrical peak (Figure 1d) which indicates homogeneity, and based on the calibration curve of Dextran standards, the molecular weights were calculated as approximately 1.76 × 10^5^ Da and 1.0 × 10^5^ Da, respectively. Analysis of the monosaccharide composition of the samples revealed that EP0-1 was composed of Man, GlcN, Glc and Gal in a ratio of 3.3:2.8:2.2:1.7, respectively (Table 1), and EP0.5-1 was composed of Man, GlcUA, Glc and Gal in a ratio of 3.0:0.7:1.5:4.8, respectively. Based on the monosaccharide composition analysis, the two polysaccharides were confirmed to be heteropolysaccharides with the neutral sugars Man, Glc and Gal as the major sugars. EP0-1 contained mostly Man, which accounted for one third of the total sugar content, while Gal was dominant in EP0.5-1. Another amino sugar GlcN was only presented in EP1-1, while EP0.5-1 had another uronic acid GlcUA. That was the key factor accounting for the ability to separate the two polysaccharides anion-exchange chromatography.

### 2.3. IR Spectroscopy Analysis

**Figure 2:**
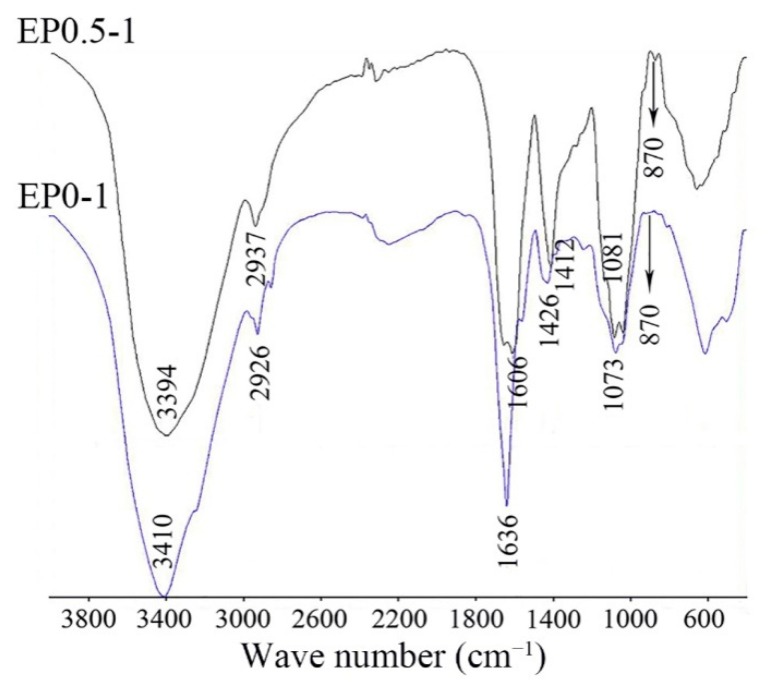
IR spectrum of EP0-1 and EP0.5-1.

Characteristic absorptions of fungal polysaccharides EP0-1, EP0.5-1 were detected in the FT-IR spectra (Figure 2), showing similar absorption bands to typical absorption peaks of the saccharide moiety.

The FT-IR spectrum of EP0-1 showed a broad and intense band corresponding to the stretching vibrations of the O–H groups of the sugar rings at 3410 cm^–1^ and the absorbance band at 2926 cm^−1^ arising from C-H stretching vibrations. The bands at 1636 cm^–1^ and 1426 cm^–1^ were assigned to the bending vibrations of O–H and C–H, respectively. The absorbance band at 1073 cm^−1^ was assigned to the stretching vibrations of C-O-C bonds in the sugars. The characteristic absorbance band at 870 cm^−1^ arose from the furan ring of sugar units in the polysaccharides. The FT-IR spectrum of EP0.5-1 showed a broad and intense band corresponding to the stretching vibrations of O–H at 3394 cm^–1^ and the absorbance band at 2937 cm^−1^ arose from C-H stretching vibrations. The bands at 1606 cm^–1^ and 1412 cm^–1^ were assigned to the bending vibrations of O–H and C–H, respectively. The absorbtion band which was near 1606 cm^–1^ and overlapped with the bending vibrations of O–H could also be C = O of carboxyl group in the uronic acid. The absorbance band at 1081 cm^−1^ was assigned to the stretching vibrations of C-O-C. The characteristic absorbance band at 870 cm^−1^ arose from furan ring of sugar units in the polysaccharides [9].

### 2.4. Analysis of Uronic Acid Reduction and Methylation

It is difficult to obtain linkage information for uronic acid by methylation and GC-MS without reducing uronic acid before it is methylated. Analysis of monosaccharide composition revealed that no uronic acid was in EP0.5-1R, which indicated the uronic acid was reduced completely.

The linkage information for samples was determined by methylation analysis (Table 2). GC-MS data revealed that EP0-1 was composed of terminal mannopyranose, (1→2)-linked mannopyranose and (1→6)-linked mannopyranose at the ratio of 1.0:4.4:2.9. Glucosamine showed only terminal linkage. Glc in EP0-1 had the linkages of terminal glucopyranose, (1→3)-linked and (1→4)-linked glucopyranose. Gal in EP0-1 had the furan configuration with the linkages of Gal*f*(1→ and →5)Gal*f*(1→ at the ratio of 1.0:4.0. EP0.5-1 had very different linkages. Gal was the dominant monosaccharide in EP0.5-1. As with EP0-1, Gal in EP0.5-1 had the furan configuration and had the linkages of Gal*f*(1→,→2)Gal*f*(1→ and →6)Gal*f*(1→ at the ratio of 1:3:2. Man in EP0.5-1 had the linkages of Man*p*(1→, →2)Man*p*(1→,→6)Man*p*(1→ and →2,6)Man*p*(1→. The →2,6)Man*p*(1→ meant EP0.5-1 was a branched polysaccharide. Glc had only two linkages with Glc*p*(1→ and →4)-Glc*p*-(1→. The linkage of →4)-Glc*p*-(1→ in uronic acid reduced sample EP0.5-1R increased to 19.8% compared with that of the normal sample EP0.5-1 at 10.6%, which indicates the GlcUA in EP0.5-1 had the 1,4 linkage.

### 2.5. NMR Spectroscopy

**Figure 3:**
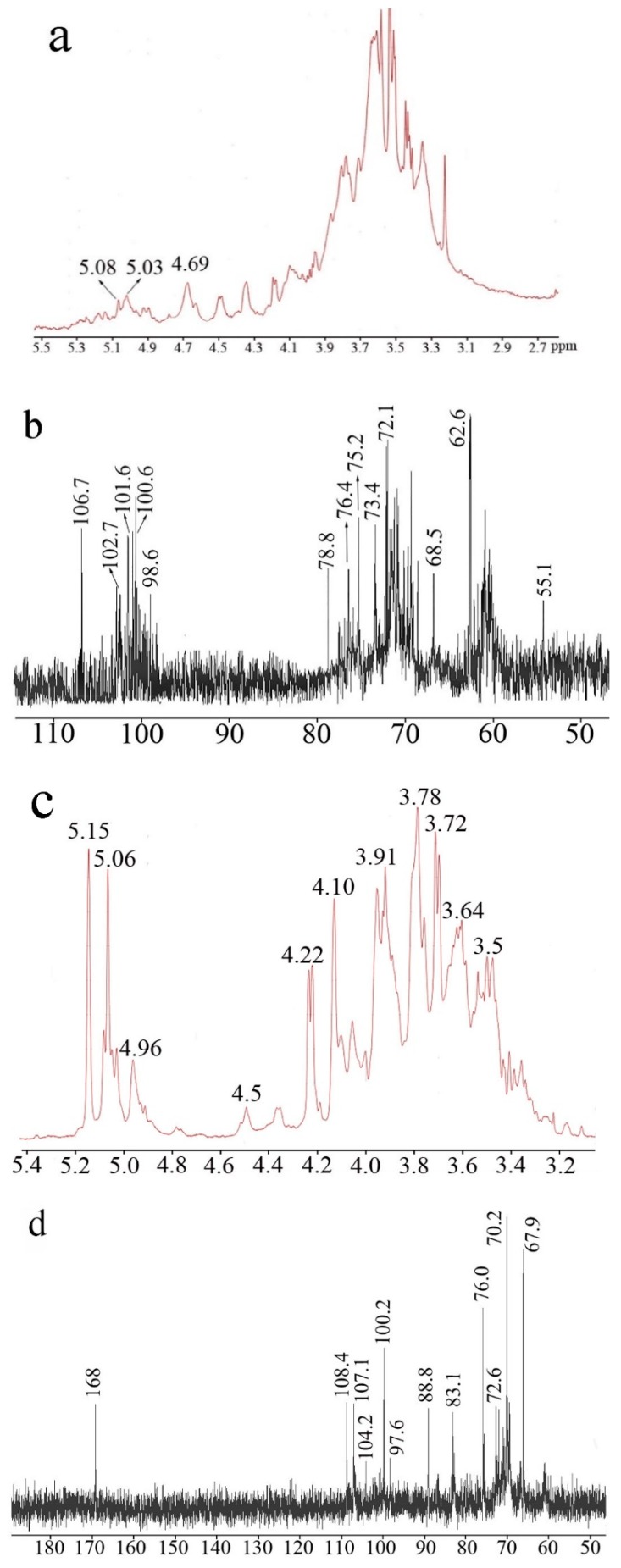
NMR spectra of EP0-1 and EP0.5-1 Spectra were performed at 25 °C on a Bruker 800-MHz NMR spectrometer using acetone as internal standard. (**a**) ^1^H NMR spectrum of EP0-1 (**b**) ^13^C NMR spectrum of EP0-1(**c**) ^1^H NMR spectrum of EP0.5-1 (**d**) ^13^C NMR spectrum of EP0.5-1.

Figure 3 shows the ^1^H- and ^13^C-NMR spectra of EP0-1 and EP0.5-1. The chemical shifts of the major signals were assigned according to reference data from similar sugar residues. The ^1^H spectrum of EP0-1 revealed that is a complex polysaccharide, evidenced by the presence of many signals in the anomeric region. The ^13^C-NMR spectrum of EP0-1 provided more characterization information. The signals at 106.7 ppm are suggested to be the anomeric carbon of (1→5)-β-d-galactofuranose due to their extremely low field shifts. The other anomeric signals were less than 103 ppm. The signals at approximately 102.7 ppm could be C1 of terminal β-GlcN and →6)-α-d-Man*p*(1→ [10]. The signal at 4.6 ppm from the ^1^H spectrum should be the anomeric proton of terminal β-GlcN(1→ [11]. The signal at 55.1 ppm was assigned to the C2 of GlcN, which is linked to the amino group. The signal at approximately 100.6 ppm was assigned to the C1 of →2)Man*p*(1→ [12]. The signal at 78.8 ppm was assigned to the substituted C2 of →2)-α-d-Man*p*(1→.

The peak at 68.5 ppm is the substituted C6 of →6)-α-d-Man*p*(1→, while the signal at 62.6 ppm represents the C6 of non-substituted mannose residues. Signals at 98.6 ppm were most likely to be α-Glc. For EP0.5-1, the signals were relatively simpler and clearer than for EP0-1. Three major anomeric signals (5.15, 5.06 and 4.96 ppm) appeared in the anomeric region. EP0.5-1 had more galactofuranose than EP0-1. Thus, the signals associated with galactofuranose were easier to distinguish. The signals at 108.4 and 107.1 ppm were assigned to the C1 of (1→2)-β-d-galactofuranose and (1→6)-β-d-galactofuranose, respectively [13,14]. The distinctive signal at 88.8 ppm was assigned to the substituted C2 of (1→2)-β-d-galactofuranose. The signal at 83.1 ppm was assigned to be C4 of β-d-galactofuranose. The signal at 67.8 ppm was assigned to the substituted C6 of →6)Gal*f*(1→, →6)Man*p*(1→, β-d-galactofuranose. EP0.5-1 contained approximately 10% GlcUA. The signal at 168 ppm was the carboxyl group signal of the GlcUA. Combined with GC-MS data, the anomeric carbon signal at 104.2 and the anomeric proton at 4.5 ppm was likely to be the anomeric signals of the (1→4)-β-d-GlcUA. Peaks at 100.2 and 97.6 ppm were most likely the signals of →2)-α-d-Man*p*(1→, and →2,6)-α-d-Man*p*(1→, respectively.

The data from structural analysis indicate that there is a clear difference between the exopolysaccharides EP0-1 and EP0.5-1 from *E. gracile*. Both polysaccharides contained the neutral sugars Man, Glc and Gal. EP0-1 also had GlcN with the linkage of terminal β-d-GlcN(1→, while EP0.5-1 contained (1→4)-β-d-GlcUA. EP0-1 had more Man, while in EP0.5-1 Gal was dominant. Man had terminal α-d-Man*p*(1→, →2)-α-d-Man*p*(1→ and →2,6)-α-d-Man*p*(1→ linkages in both polysaccharides, but EP0.5-1 had an additional →2, 6)-α-d-Man*p*(1→ linkage, which indicates that it is a branched polysaccharide. A remarkable characteristic of Gal in EP0-1 and EP0.5-1 is that the galactose is in the form of a galactofuranose (β-d-Gal*f*) furan ring. EP0-1 mainly had the (1→5)-β-d-Gal*f* linkage. EP0.5-1 had (1→2)-β-d-Gal*f* and (1→6)-β-d-Gal*f* in higher proportion [15]. Galactofuranose has been found in many fungi [16,17]. Polysaccharides that contain galactofuranose are diverse in structure and are mostly reported in the species of *Penicillium* and *Aspergillus*. (1→5)-β-d-Gal*f* was the main linkage of galactofuranose in these exopolysaccharides. In the exopolysaccharides of *E. gracile*, the galactofuranose showed high diversity with the linkages of terminal β-d-Gal*f*, (1→2)-β-d-Gal*f*, (1→5)-β-d-Gal*f* and (1→6)-β-d-Gal*f*, which could be the characteristics of the exopolysaccahrides in the genus *Exobasidium*.

### 2.6. Determination of Macrophage Activation Activity

The modified *C. oleifera* leaves in this study were infected by *E. gracile*, and produced many different polysaccharides that are comparable in features with those resulting from black tea processing. Since bioactive heteropolysaccharides have been found in black tea, the exopolysaccharides in *E. gracile* were screened for macrophage activation activity to assess the potential functions of fungal polysaccharides in the *C. oleifera* leaves.

**Figure 4:**
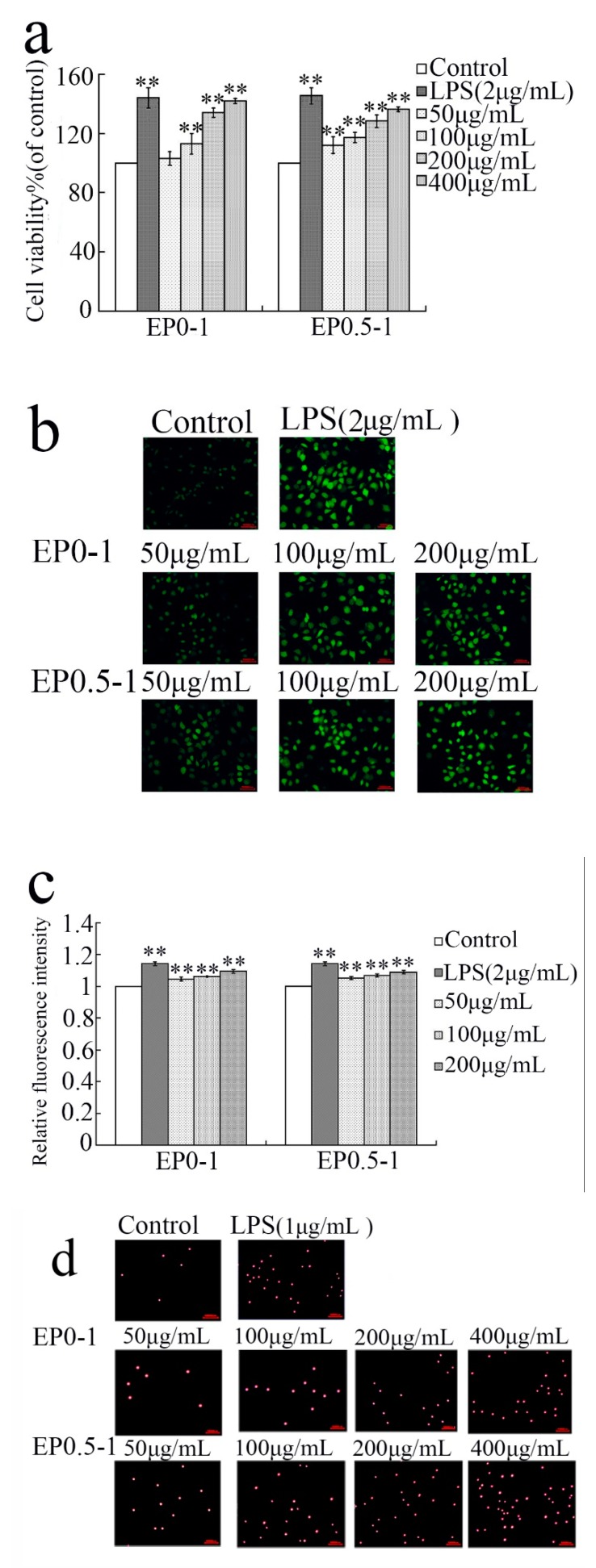

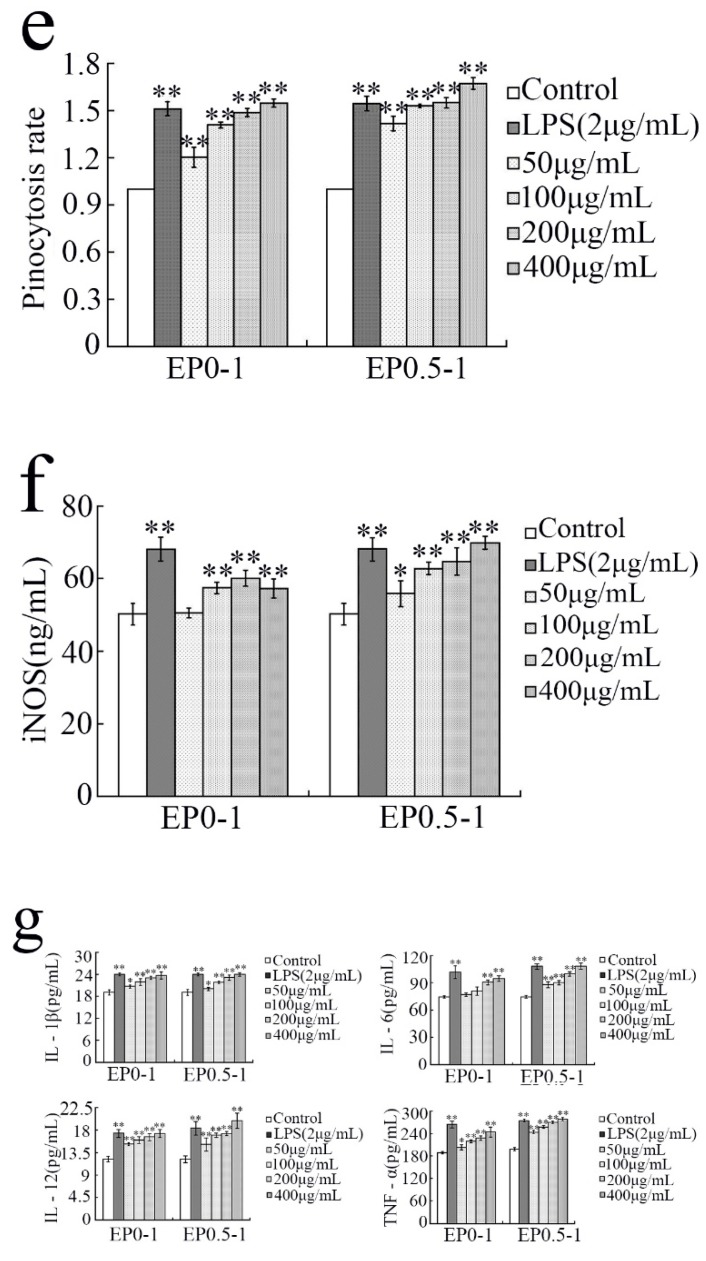
Immunomodulatory activity of EP0-1 and EP0.5-1 in RAW264.7 macrophages. (**a**) Effects of EP0-1 and EP0.5-1 on RAW 264.7 cell proliferation. (**b**) Effect of EP0-1 and EP0.5-1on ROS fluorescence. (**c**) ROS production. (**d**) RAW264.7 cells phagocytosed endocytic fluorescent-red latex beads (red fluorescence). (**e**) Phagocytotic activity of RAW 264.7 cells stimulated by EP0-1 and EP0.5-1. (**f**) The effect of EP0-1 and EP0.5-1 with different concentrations and LPS on the levels of iNOS in RAW264.7. (**g**) The effect of EP0-1 and EP0.5-1 with different concentrations and LPS on the levels of IL-1β, IL-6, IL-12 and TNF-α in RAW264.7. The results were expressed as means ± SD (*n* = 3). *P < 0.05, **P < 0.01 vs. control group.

After treatment with different concentrations of LPS, EP0-1 and EP0.5-1 for 24 h, cells were assessed for viability using an MTT assay. The viability effects of LPS, EP0-1 and EP0.5-1 on RAW 264.7 cells are shown in Figure 4a. EP0-1 and EP0.5-1 were not cytotoxic to RAW264.7 cells. Compared with the control, EP0-1 had significant proliferation activity at the concentration range of 100–400 μg/mL. At the concentration range of 50–400 μg/mL, EP0.5-1 significantly promoted the proliferation rate of macrophages.

Reactive oxygen species (ROS) are produced by the activation of macrophages and are an important molecule in signaling pathways that are involved in the immune response. ROS plays a positive role in preventing and killing microbial invasion and tumor genesis [18]. The production of ROS can be measured using the fluorescent probe DCFH-DA. The cell was probed with DCFH-DA after treated with different concentrations of LPS, EP0-1 and EP0.5-1 for 24 h. The mean fluorescence intensity of DCF in LPS, EP0-1 and EP0.5-1 treated cells was significantly greater than the control, based on fluorescence microscopy results (Figure 4b). This result demonstrated that the cells treated with EP0-1 and EP0.5-1 in the concentration range of 50–200 μg/mL displayed up-regulation of intracellular ROS production (Figure 4c).

The effect of EP0-1 and EP0.5-1 on the pinocytic activity of RAW264.7 cells was examined by the neutral red uptake assay. The cells visibly increased in cells treated with EP0-1 and EP0.5-1 compared with the normal control (Figure 4d). The result indicates that macrophages treated with EP0-1 and EP0.5-1 exhibit strong pinocytic activity. EP0-1 and EP0.5-1 exhibited significant macrophage activation activity within the concentration range of 50–400 μg/mL (Figure 4e).

NO has proven to be an important messenger molecule. The release of NO by macrophages regulates the inflammatory response and can activate functions of the immune system. Therefore, NO is the main effector molecule after macrophage activation. When macrophages are activated, they can express iNOS, and iNOS is the most important enzyme that catalyzes NO production [19,20]. Effects on the iNOS secretion by macrophages were observed at EP0-1 concentrations ranging from 100–200 μg/mL, which gradually increased iNOS concentrations; EP0.5-1 concentrations ranging 50-400 μg/mL, increased iNOS concentrations in a dose-dependent manner (Figure 4f).

Cytokines are small molecular weight proteins secreted by activated monocyte-macrophages and lymphocytes that can regulate the immune response. IL-1β, IL-6, IL-12 and TNF-α are important cytokines produced by activated macrophages [21]. EP0-1 and EP0.5-1 increased IL-1β, IL-12 and TNF-α production in a dose-dependent manner when applied at 50–400 μg/mL. Concentrations of EP0-1 increasing from 200-400 μg/mL were associated with a gradual increase in IL-6 concentrations. EP0.5-1 concentrations ranging from 50-400 μg/mL were associated with an increase in IL-12 concentrations in a dose-dependent manner (Figure 4g) (*P* < 0.05).

Macrophages play an important role in host defense systems against microbial infections and tumors. Under normal conditions, macrophages remain in a static state, but when the body is stimulated by pathological material or injury, macrophages can activate and destroy foreign matter indirectly via the secretion of macrophage-derived biological factors. Activated macrophages can phagocytose and destroy targeted organisms through enhancing the release of NO, iNOS, ROS, and several cytokines, such as IL-1β, IL-6, IL -12, TNF-α [22,23]. Macrophages were selected as model to investigate the immunoregulation effect of EP0-1 and EP0.5-1 due to their important role in the immune system. Results showed that EP0-1 and EP0.5-1 not only improved the proliferation and phagocytosis of macrophages, but also induced the secretion of NO, iNOS, ROS, and several cytokines, such as IL-1β, IL-6, IL-12 and TNF-α, which increased in concentration in a dose-dependent manner. In addition, EP0.5-1 at 400μg/mL significantly increased the production of these cytokines, especially TNF-α, IL-12 [24,25] (*P* <0.05).

## 3. Materials and Methods

### 3.1. Fungal Isolation and Culture

Healthy *Camellia oleifera* Abel. leaves and infected leaves were obtained from Hunan Province, China. *Exobasidium gracile* was isolated from fresh infected leaves of *C. oleifera*. Typical and well-grown infected leaves without signs of infection from other fungi were cut to 5 mm × 5 mm pieces using a sterilized scalpel. Leaf pieces with foliage outward were fixed to the lid of the upright Petri dish using double sided tape. PDA agar medium was poured into the Petri dish, and was incubated at 25 °C to allow the spores to diffuse on to the medium. After 12-24 h, the lid was changed to a sterilized lid and the agar containing spores was cultivated upside down. Pure culture of *E. gracile* was obtained after 5 days. *Exobasidium gracile* mycelia were inoculated into PDA liquid medium and cultured for one month at 25 °C [4].

### 3.2. Extraction and Purification of Fungal Exopolysaccharides

Mycelial biomass and culture supernatant of *E. gracile* were separated using vacuum filtration. The supernatant was concentrated using reduced pressure, and ethanol (5 times the volume of concentrated solution) was added to form a precipitate by incubating at 4 °C for 24 h. After centrifugation (4000 r/min, 15 min), precipitate was collected then dialyzed (Dialysis bag: MD55-3500-05, Union Carbide Corporation Co., Greensburg, LA USA) against deionized water for 48 h at room temperature. Finally, the sample was lyophilized to obtain crude polysaccharides (ECP).

The ECP samples were fractionated using anion exchange chromatography by a Q Sepharose Fast Flow column (300 × 30 mm) with water and NaCl solution as the mobile phase. First, the samples were eluted with a linear elution gradient of 0–2 mol/L NaCl solution. Fractions were then assayed for carbohydrate content using the phenol–sulfuric acid method. According to the linear gradient elution results, gradient elution was used to separate the polysaccharides into two fractions, which were eluted with distilled water and 0.5 mol/L NaCl separately. The major fractions eluted by each gradient were pooled, dialyzed and further purified on a Superdex 75 column (100 × 2 cm), which was eluted with 0.2 mol/L NH_4_HCO_3_ solution at a flow rate of 0.3 mL/min. The major polysaccharide fractions were pooled, freeze-dried and named EP0-1 and EP0.5-1.

### 3.3. Analysis of Physicochemical Characteristics

The purity and molecular weight of polysaccharides were determined by high-performance gel permeation chromatography (HPGPC) with a TSKgel G3000PWXL column (7.8 mm × 30.0 cm, Tosoh, Tokyo, Japan). The detection conditions were as follows: refractive index detector (Agilent 1100 Series); mobile phase was 0.2 mol/L Na_2_SO_4_ solution; flow rate at 0.5 mL/min; temperature was 35 °C. Twenty μL of 1% sample solutions in 0.2 mol/L Na_2_SO_4_ were injected. The molecular weight was estimated by referencing to a calibration curve made from a set of Dextran T-series standards (Mw: 70.8, 34.4, 20.0, 10.7, 4.71, 2.11, 9.6 and 5.9 kD).

Total sugar contents were determined by phenol–sulfuric acid method using glucose as the standard. The monosaccharide compositions were analyzed by 1-phenyl-3-methyl-5-pyrazolone (PMP) pre-column derivatizated HPLC and UV detection. Sugar identification was done by comparison with reference sugars (l-rhamnose (Rha), l-arabinose (Rha), l-fucose (Fuc), d-xylose (Xyl), d-mannose (Man), d-galactose (Gal), d-glucose (Glc), d-glucuronic acid (GlcUA), d-galacturonic acid (GalUA), and N-acetyl-d-glucosamine (GlcNAc). Calculation of the molar ratio of the monosaccharide was carried out on the basis of the peak area of the monosaccharide. [9] The protein content was determined by Lowry method using bovine serum albumin as standard [26]. The sulfate content was determined by the barium chloride-gelatin method using K_2_SO_4_ as standard. The uronic acid content was determined by the carbazole-sulfuric acid method using glucuronic acid as standard [27]. The amino sugar content was determined by the ehrlich method using glucosamine as standard.

### 3.4. IR Spectroscopy Analysis

The IR spectra were measured using a Nicolet NEXUS 470FT-IR spectrometer (Thermo Fisher Co., Waltham, MA USA). A small amount of polysaccharide (2 mg) was mixed with KBr powder, ground, then pressed into a 1 mm pellets and scanned 32 times using FT–IR with measurement in the frequency range of 4000–500 cm^–1^. The instrument worked with a resolution ratio of 4.0 cm^–1^. The FT–IR spectrum of the polysaccharide was measured using the Nicolet Omnic software [28].

### 3.5. Uronic acid Reduction

EP0.5–1 had a very evident uronic acid content. To analyze the linkages of uronic acid, EP0.5-1 was subjected to uronic acid reduction. The sample was converted from a sodium type to hydrogen type by a 732 cation exchange resin, and then 5 mg of hydrogen type sugar was dissolved into 2 mL of 67% tetrahydrofuran (THF)—0.3 moL/L 2-(4-morpholino)ethanesulfonic acid (MES) buffer solution and adjust the pH to 4.75 with 10% triethylamine. After sufficient dissolution, the mixture was added 1-ethyl-3-(3-dimethylaminopropyl) carbodiimide (EDC, 20 mg) and stirred for 1 h at room temperature. Next, 2 mL of freshly prepared 2 mol/L NaBH_4_ was added and stirred for another 1 h at 50 °C. Excessive NaBH_4_ was decomposed with HOAc [29,30,31]. The mixture was dialyzed against deionized water for 48 h at room temperature and lyophilized to obtain uronic acid reduced polysaccharides (EP0.5-1R). Monosaccharide composition analysis was used to determine the content of uronic acid.

### 3.6. Methylation Analysis

Methylation analysis was carried out according to the method of modified Hakomori method. Polysaccharides (EP0-1, EP0.5-1) and uronic acid reduced polysaccharides (EP0.5-1R) respectively were dissolved in dimethyl sulfoxide (DMSO) and methylated with a suspension of sodium hydride (NaH)/DMSO and iodomethane. The reaction mixture was extracted with dichloromethane, the solvent was removed by vacuum evaporation, and then the methylated sample was hydrolyzed as described above, reduced with sodium borodeuteride and converted into partially methylated alditol acetates and analyzed by GC-MS on a HP6890II/5973 instrument using a DB-5ms fused silica capillary column (0.25 mm × 30 m) (Agilent Technologies Co., Foster City, CA, USA) [32,33,34,35]. The detection conditions were as follows: Temperature was increased from 100 °C to 220 °C flow rate of 5 °C/min then maintained at 220 °C for 5 min with helium as carrier gas. The peaks on the chromatogram were identified from their retention times and the mass fragmentation patterns.

### 3.7. NMR Spectroscopy

Freeze-dried EP0-1 and EP0.5-1(50 mg) were dissolved in 400 μL of D_2_O (99.96 atom%) and lyophilized two times to exchange protons and transferred to a 5 mm NMR tube. ^1^H- and ^13^C-NMR were recorded on a Bruker 800-MHz NMR spectrometer (Bruker Biospin Co., Billerica, MA, USA) and acquisition of the spectra was carried out using Topspin 2.1.6 software. All spectra were acquired at a temperature of 298 K [36,37]. For ^1^H spectrum, the number of scans was 24; Spectral width was 13.886 ppm; Acquisition time was 1.4746 s. For ^13^C spectrum, the number of scans was 10240; Spectral width was 238.922; Acquisition time was 0.69999 s.

### 3.8. Determination of Macrophage Activation Activity

#### 3.8.1. Cell Viability Assay

The effect of purified polysaccharide on the viability of RAW264.7 cells were analyzed using the 3-(4, 5-dimethylthiazol-2-yl)-2, 5-diphenyltetrazolium bromide (MTT) assay. The RAW264.7 cells were purchased from the Type Culture Collection of Chinese Academy of Sciences (Shanghai, China) and pre-incubated in 96-well plate at density of 1 × 10^6^ cells mL^−1^ per well for 24 h and then were respectively cultured with LPS (2 μg/mL) and different concentrations of EP0-1 and EP0.5-1 for 24 h. After incubation, the cells were stained with MTT (30 μL/well) to a final concentration of 0.5 mg mL^−1^ for 4h at 37 °C in dark, and then the supernatants were removed before adding DMSO (100 μL/well). Finally, the absorbance was measured at 570 nm by microplate ELISA reader [21].

#### 3.8.2. Measurement of ROS Generation

ROS production by RAW264.7 was measured using the fluorescent probe DCFH-DA (2,7-dichlorodihydrofluorescein diacetate). The RAW264.7 cells were seeded at 5 × 10^5^ cells/well in glass bottom cell culture dishes overnight. LPS (2 μg/mL), and the various concentrations of polysaccharides (50, 100, 200 μg/mL) were added into each well, and these cells were incubated for 24 h at 37 °C. And then all the culture medium were removed before adding DCFH-DA (10 μL/well) for 30 min at 37 °C. Finally, the cells were washed twice with PBS. The degree of fluorescence was detected at 485 nm excitation and at 535 nm emission using fluorescence microscopy [18].

#### 3.8.3. Determination of Phagocytic Uptake

The phagocytic activity of the macrophages was determined using the fluorescent-red latex beads (2 μm) and neutral red. RAW264.7 cells were seeded at 1 × 10^4^ cells well ^−1^ in a 96-well plate and incubated at 37 °C in a humidified atmosphere containing 5% CO_2._ After 24 h, DMEM medium, LPS (2 μg/mL) or the various concentrations of polysaccharides (50, 100, 200, 400 μg/mL) were added into each well, and these cells were incubated for 24 h at 37 °C. Each concentration was repeated three wells. Culture media were removed and 0.1% latex beads (red fluorescent, excitation/emission of 553/635 nm) or 0.075% neutral red was added, and incubated for 30 min. The cells were washed twice with phosphate-buffered saline (PBS). For analysis of cell phagocytic uptake of fluorescent red-aqueous suspension, the cells were stained with MitoTracker (mitochondrial, green, excitation/emission of 490/516 nm) for 15 min. The phagocytic activity of RAW264.7 cells was determined using laser-scanning confocal microscopy (FV 1000, Olympus Co., Tokyo, Japan) after staining with MitoTracker. For analysis of cells phagocytic uptake of the neutral red, cell lysing solution (150 μL/well) were added into each well and cells were cultured for 1 h at 37 °C. The absorbance was evaluated in an ELISA reader at 570 nm. $\mathrm{Pinocytic}\;\mathrm{ability}\;\left(\%\right)=\left(\frac{\mathrm{Asample}}{\mathrm{Acontrol}}-1\right)\times 100\%$, where A_control_ was the absorbance of control without the tested samples, and A_sample_ is the absorbance in the presence of the tested samples [18].

#### 3.8.4. Measurement of Inducible Nitric Oxide Synthase (iNOS) Production

The RAW264.7 cells were pre-incubated in 96-well plate at density of 1 × 10^6^ cells/mL per well for 24h. Then, LPS (2 μg mL^−1^) as the positive control and the various concentrations of polysaccharides were added into each well, and these cells were incubated for 24 h at 37 °C, cell culture supernatants were collected, and the iNOS level was determined by ELISA kit (Shanghai mlbio Biotechnology Co. Ltd., Shanghai, China), according to the manufacturer’s instructions [38].

#### 3.8.5. Cytokine Assays

The RAW264.7 cells were pre-incubated in 96-well plate at density of 5 × 10^5^ cells/mL per well for 24 h with LPS (2 μg/mL) and the various concentrations of polysaccharides as above. After 24 h of treatment, cell culture supernatants were collected by centrifugation at 1000 rpm for 8 min at 4 °C and stored at −20 °C. Finally, analyzed the productions of IL-1β, IL-6, IL-12 and TNF-α by using ELISA kit (Shanghai mlbio Biotechnology Co. Ltd.), according to the manufacturer’s instructions [39].

#### 3.8.6. Statistical Analysis

All data obtained in this study were processed statistically and divergences were presented as mean ± SD. SPSS 16.0 for Windows (Pearson Co., Beijing, China) was used and *P* < 0.05 indicated significance differences.

## 4. Conclusions

A previous study revealed the total sugar content, reducing sugars, crude polysaccharide, and the total phenol contents all increased in the infected leaves after infection by the parasitic fungus *E. gracile*. The crude polysaccharides from infected leaves had stronger antioxidant activity than those of the healthy leaves. Monosaccharide composition analysis indicates the exopolysaccharides were present in the crude polysaccharides from infected leaves.

Based on the tea polysaccharides of black tea, which is also fermented by microorganisms, the parasitic fungus in the infected leaves from *E. gracile* was isolated, purified and fermented. The exopolysaccharides in the fermentation broth were purified. Two heteropolysaccharides (EP0-1 and EP0.5-1) were obtained. EP0-1 was found to be an alkaline polysaccharide that mainly has the linkages of α-d-Man*p*(1→, →2)-α-d-Man*p*(1→ and →6)-α-d-Man*p*(1→, →3)-α-d-Glc*p*(1→ and →4)-α-d-Glc*p*(1→, terminal β-d-Gal*f*, (1→5)-β-d-Gal*f*, and terminal β-d-GlcN(1→. EP0.5-1 was an acidic and galactofuranose-containing polysaccharide. It was found to contain the linkages α-d-Man*p*(1→, →2)-α-d-Man*p*(1→, →6)-α-d-Man*p*(1→,→2, 6)-α-d-Man*p*(1→, →4)-α-d-Glc*p*(1→, and →4)-α-d-GlcUA(1→. The linkages of galactofuranose were composed of terminal β-d-Gal*f*, (1→6)-β-d-Gal*f* and (1→2)-β-d-Gal*f*. Preliminary activity tests suggest that the exopolysaccharides from *E. gracile* have significant immunoregulatory activity by activating macrophages.

The results indicate that the polysaccharides produced by *E. gracile* show potential immunoregulatory activity. Thus, the infected leaves of *C. oleifera* are worthy of further investigation for use as a functional tea beverage.

## Figures and Tables

**Table 1:** Monosaccharide composition of EP0-1 and EP0.5-1.

Sample	Monosaccharide Composition (% m/m)				
	Man	GlcN	GlcUA	Glc	Gal
EP0-1	3.3	2.8	-	2.2	1.7
EP0.5-1	3.0	-	0.7	1.5	4.8

**Table 2:** Methylation analysis of EP0-1, EP0.5-1, EP0.5-1R.

Methylation Product	Linkage Type	Main MS (*m/z*)	Molar Ratio (100%)		
			EP0-1 EP0.5-1 EP0.5-1R		
1,5-Ac_2_-2,3,4,6-Me_4_-d-Man	Man*p*(1→	101,117,129,145,161,205	4.9	7.8	9.0
1,5-Ac_-_2-3,4,6-Me_3_-d-GlcN	GlcN*p*(1→	101,117,129,145,159,203	14.8		
1,5-Ac2-2,3,4,6-Me4-d-Glc	Glc*p*(1→	101,117,129,145,161,205	4.4	6.9	4.5
1,4-Ac_2_-2,3,5,6-Me_4_-d-Gal	Gal*f*(1→	101,117, 161,205,277	4.1	9.0	3.3
1,2,5-Ac_3_-3,4,6-Me_3_-d-Man	→2)Man*p*(1→	101,117,129,145,161,189	21.7	10	8
1,3,5-Ac_3_-2,4,6-Me_3_-d-Glc	→3)Glc*p*(1→	101,117,129,161,233	11.7		
1,4,5-Ac_3_-2,3,6-Me_3_-d-Glc	→4)-Glc*p*-(1→	113,117,131,161,173,233	7.5	10.6	19.8
1,4,5-Ac_3_-2,3,6-Me_3_-d-Gal	→5)-Gal*f*-(1→	113,117,131,161,173,233	16.3		
1,2,4-Ac_3_-3,5,6-Me_3_-d-Gal	→2)Gal*f*(1→	101,117,129,143,161		19.2	17
1,4,6-Ac_3_-2,3,5-Me_3_-d-Gal	→6)Gal*f*(1→	101,117,127,159,233		14	12.7
1,5,6-Ac_3_-2,3,4-Me_3_-d-Man	→6)Man*p*(1→	101,117,129,161,189	14.3	9	7.8
1,2,5,6-Ac_4_-3,4-Me_2_-d-Man	→2,6)Man*p*(1→	129,189		13.5	17.9

## Supplementary Materials

The following are available online at https://www.mdpi.com/1420-3049/24/11/2048/s1, Figure S1: The free radical scavenging activities of the extracts and the polysaccharides from the normal leaves and the modified leaves. Table S1: General content of the sugar-related components from the extract of the normal and the modified leaves in *Camellia oleifera*. Table S2: Monosaccharide composition of leaves extract and the crude polysaccharides.
