# Supplementary material for: Bioactive Exopolysaccharides Reveal Camellia oleifera Infected by the Fungus Exobasidium gracile Could Have a Functional Use
Source: Molecules. 2019 May 29;24(11):2048. doi: 10.3390/molecules24112048 (PMC6600561; doi:10.3390/molecules24112048)
Supplement: Supplementary file 1 [file molecules-24-02048-s001.pdf]

The properties of sugar related components were compared between the normal leaves and the modified leaves of *Camellia oleifera*. For the modified leaves, the total sugar content increased to 45% compared with the normal leaves at 31.4%. The reducing sugar was also significantly increased to 27.8% from 5%. It could explain the reason that the modified leaves tasted sweeter. And there was obvious difference in polysaccharides content between the normal leaves and the modified leaves. The yields of the crude polysaccharide in ME was 16.9%, while that of CE was 10.24% It is worth mentioning that the total phenol contents of ME was also increased to 26.9%. The comparison of the sugar-related components from the normol and modified leaves was showed in Table 1.

The monosaccharide composition analysis of CE, ME, CEP and MEP showed the sugar composition of the leaves had changed dramatically (Table 2). CE mainly contained Glc, Rha Gal and Arb which was similar with the tea polysaccharide. ME which was from the infectious leaves was composed of more Man, Glc and Gal but less Arb. The polysaccharides of the leaves had the same variation tendency. Compared with the total sugar, the polysaccharide CEP had more Gal and Arb. MEP had more Man and Gal but no Arb. Man, Glc and Gal were common in the fungus polysaccharides. The increase of Man and Gal in MEP, the polysaccharide from the infectious leaved might be due to the exopolysaccharide from the fungus. The results also indicated that the *Exobasidium gracile could* promote the degradation of polysaccharide such as cellulose in the leaves into some substances of energy source. It could also explain the reason that the reducing sugar increased in the fungus infected leaves ME.

Table 1 General content of the sugar-related components from the extract of the normal and the modified leaves in *Camellia oleifera*

Contents (%)	Sample	
	CE	ME
Total sugar	31.4±1.41	45±2.4
Reducing sugar	5.1±1.7	27.8±1.2

Total phenol	22.9±0.26	26.9±0.16
Polysaccharide	10.24±1.1	16.9±1.3

CE means extract from healthy leaves of *Camellia oleifera*, ME means infected leaves extract.

Table 2 Monosaccharide composition of leaves extract and the crude polysaccharides.

Sample	Monosaccharide composition (% m/m)				
	Man	Rha	Glc	Gal	Arb
CE	0.7	11.7	82.8	2.4	2.4
ME	4.4	2.5	85.6	5	2.5
CEP	4	11	46	21	18
MEP	27	0	41	25	7

CE means extract from healthy leaves of *Camellia oleifera*, ME means infected leaves extract.CEP means polysaccharides from healthy leaves extract of *Camellia oleifera*. MEP means polysaccharides from infected leaves extract.

The tea extract had excellent antioxidant activity. Thus, the extracts from the normal leaves, the modified leaved and the polysaccharides were investigated the free radical scavenging activity. The DPPH radical scavenging activity of CE, ME, CEP and MEP were carried out and compared with ascorbic acid, a standard antioxidant. The radical scavenging abilities of the samples on DPPH radicals concentration-dependent. But the leaves extracts had better DPPH radical scavenging activity. The EC_{50} of CE and ME for the DPPH radical scavenging were 0.07mg/mL and 0.03mg/ml, respectively. The leaves extract had good antioxidant activity, which may be related to the rich phenolic substances. The EC_{50} of the polysaccahrides CEP and MEP was 4.7 mg/ml and 3.4 mg/mL, respectively (Fig.a-b). The DPPH radical scavenging activity of ME and MEP from the modified leaves were both better than CE and CEP from the normal leaves. It indicated the infection of *Exobasidium gracile* to the leaves coule promote the DPPH radical scavenging activity.

The Hydroxyl radical scavenging activity of CE and ME with CEP and MEP were compared. The EC_{50} of CE and ME for the hydroxyl radical scavenging were 0.5mg/mL and 0.3mg/mL, respectively. CEP and MEP have EC_{50} of 8.7mg/ml and 7.4 mg/mL (Fig.c-d). The same as the DPPH radical scavenging activity, the *Exobasidium gracile* infection can increase the hydroxyl radical scavenging activity.

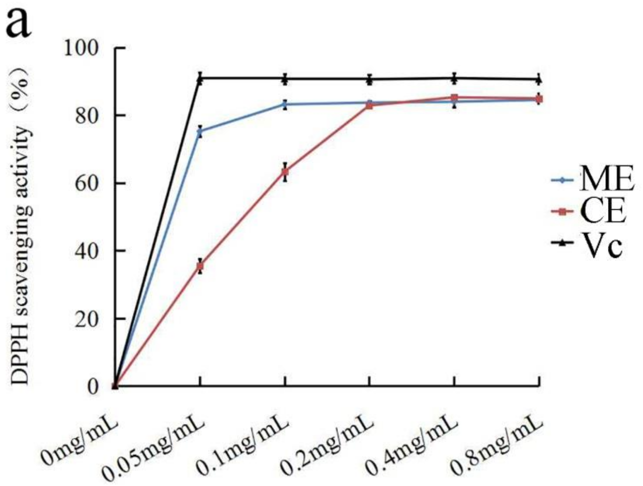

a. DPPH scavenging activity comparison of ME and CE.

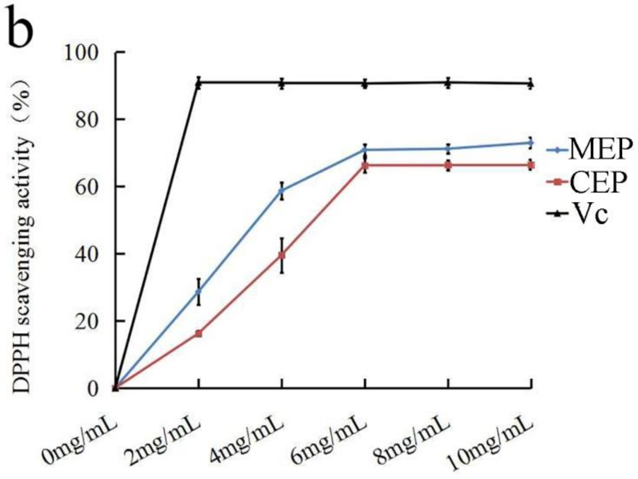

b. DPPH scavenging activity comparison of MEP and CEP

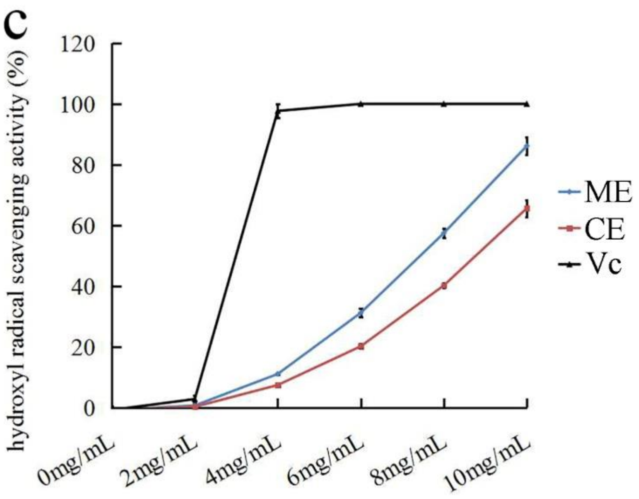

c. Hydroxyl radical scavenging activity comparison of ME and CE

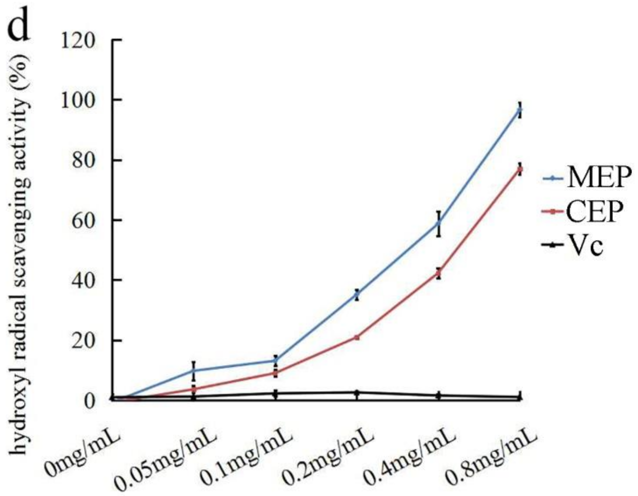

d. Hydroxyl radical scavenging activity comparison of MEP and CEP
